# PSTPIP2 inhibits cisplatin-induced acute kidney injury by suppressing apoptosis of renal tubular epithelial cells

**DOI:** 10.1038/s41419-020-03267-2

**Published:** 2020-12-12

**Authors:** Hong Zhu, Wenjuan Jiang, Huizi Zhao, Changsheng He, Xiaohan Tang, Songbing Xu, Chuanting Xu, Rui Feng, Jun Li, Taotao Ma, Cheng Huang

**Affiliations:** Inflammation and Immune Mediated Diseases Laboratory of Anhui Province, Anhui Institute of Innovative Drugs, School of Pharmacy, Anhui Medical University, Hefei, 230032 China

**Keywords:** Cancer, Cancer prevention

## Abstract

Cisplatin (CP) is an effective chemotherapeutic agent widely used in the treatment of various solid tumours. However, CP nephrotoxicity is an important limitation for CP use; currently, there is no method to ameliorate cisplatin-induced acute kidney injury (AKI). Recently, we identified a specific role of proline–serine–threonine phosphatase-interacting protein 2 (PSTPIP2) in cisplatin-induced AKI. PSTPIP2 was reported to play an important role in a variety of diseases. However, the functions of PSTPIP2 in experimental models of cisplatin-induced AKI have not been extensively studied. The present study demonstrated that cisplatin downregulated the expression of PSTPIP2 in the kidney tissue. Administration of AAV-PSTPIP2 or epithelial cell-specific overexpression of PSTPIP2 reduced cisplatin-induced kidney dysfunction and inhibited apoptosis of renal tubular epithelial cells. Small interfering RNA-based knockdown of PSTPIP2 expression abolished PSTPIP2 regulation of epithelial cell apoptosis in vitro. Histone acetylation may impact gene expression at the epigenetic level, and histone deacetylase (HDAC) inhibitors were reported to prevent cisplatin-induced nephrotoxicity. The UCSC database was used to predict that acetylation of histone H3 at lysine 27 (H3K27ac) induces binding to the PSTPIP2 promoter, and this prediction was validated by a ChIP assay. Interestingly, an HDAC-specific inhibitor (TSA) was sufficient to potently upregulate PSTPIP2 in epithelial cells. Histone acetylation-mediated silencing of PSTPIP2 may contribute to cisplatin nephrotoxicity. PSTPIP2 may serve as a potential therapeutic target in the prevention of cisplatin nephrotoxicity.

## Introduction

Cisplatin is one of the most generally used and highly effective chemotherapy drugs for the treatment of various types of human cancer, such as testicular, ovarian, head and neck, bladder, bone, muscle, small and non-small cell lung and cervical cancers, sarcomas and lymphomas^1,2^. Unfortunately, the clinical application of cisplatin is severely limited by considerable side effects associated with nephrotoxicity, which is one of the most common side effects of cisplatin^3,4^. The existing therapy for cisplatin nephrotoxicity in humans does not completely prevent AKI occurrence^5,6^. Therefore, there is an urgent need to find an effective therapy based on the mechanism of cisplatin nephrotoxicity.

Apoptosis of tubular epithelial cells and renal inflammation have been shown to be a prominent and characteristic feature of cisplatin-induced AKI^7,8^. Recent studies suggested that proline–serine–threonine phosphatase-interacting protein 2 (PSTPIP2) has immunomodulatory activity and is closely associated with cell proliferation and apoptosis^9–11^. PSTPIP2 is a member of the Pombe Cdc15 homology (PCH) family of proteins located on chromosome 18 and is known as a protein important for the organisation of the cytoskeleton at the cell membrane^12,13^. PSTPIP2 is expressed in various organs and tissues, such as brain, heart, kidney, lung, liver, etc. At the cellular level, the expression of PSTPIP2 was detected in monocytes, lymphocytes, granulocytes and mast cells. PSTPIP2 plays an imperative role in tumours, autoimmune diseases and other diseases by influencing cell proliferation, apoptosis and secretion of inflammatory factors^14–18^. Ales Drobek et al. showed that PSTPIP2 binds lipid phosphatase SHIP1 and protein tyrosine kinase Csk (negative regulators of inflammation) to prevent the development of auto-inflammation. Baum et al. reported that PSTPIP2 can bind and interact with the intracellular FasL domain, regulate FasL localisation in the cytoplasm, and coordinate apoptosis of HEK 293T, COS-1, RBL2H3 and other cells. Moreover, Halle et al. found that PSTPIP2 controls the function of activated caspase-3 by acting as a substrate of PTP-PEST and plays an important role in cell apoptosis^10,19,20^. These studies suggest that investigation of the effect of PSTPIP2 on apoptosis of renal tubular epithelial cells can promote the understanding of the pathogenesis of cisplatin-induced AKI and provide ideas for clinical prevention and treatment of cisplatin-induced AKI to identify new drug targets.

In this study, we investigated whether PSTPIP2 can suppress cisplatin nephrotoxicity and what are the mechanisms of this effect. Our results show that administration of AAV-PSTPIP2 or epithelial cell-specific overexpression of PSTPIP2 reduces cisplatin-induced kidney dysfunction and inhibits apoptosis of renal tubular epithelial cells. Interestingly, we have detected that histone H3 acetylated at lysine 27 (H3K27ac) binds to the PSTPIP2 promoter. Furthermore, an HDAC inhibitor trichostatin A (TSA) can upregulated PSTPIP2 in vivo and in vitro. In conclusion, this study is the first to demonstrate that PSTPIP2 plays a pivotal role in AKI and may be a potential therapeutic target for cisplatin nephrotoxicity.

## Results

### PSTPIP2 was downregulated in vivo and in vitro

**Fig. 1:**
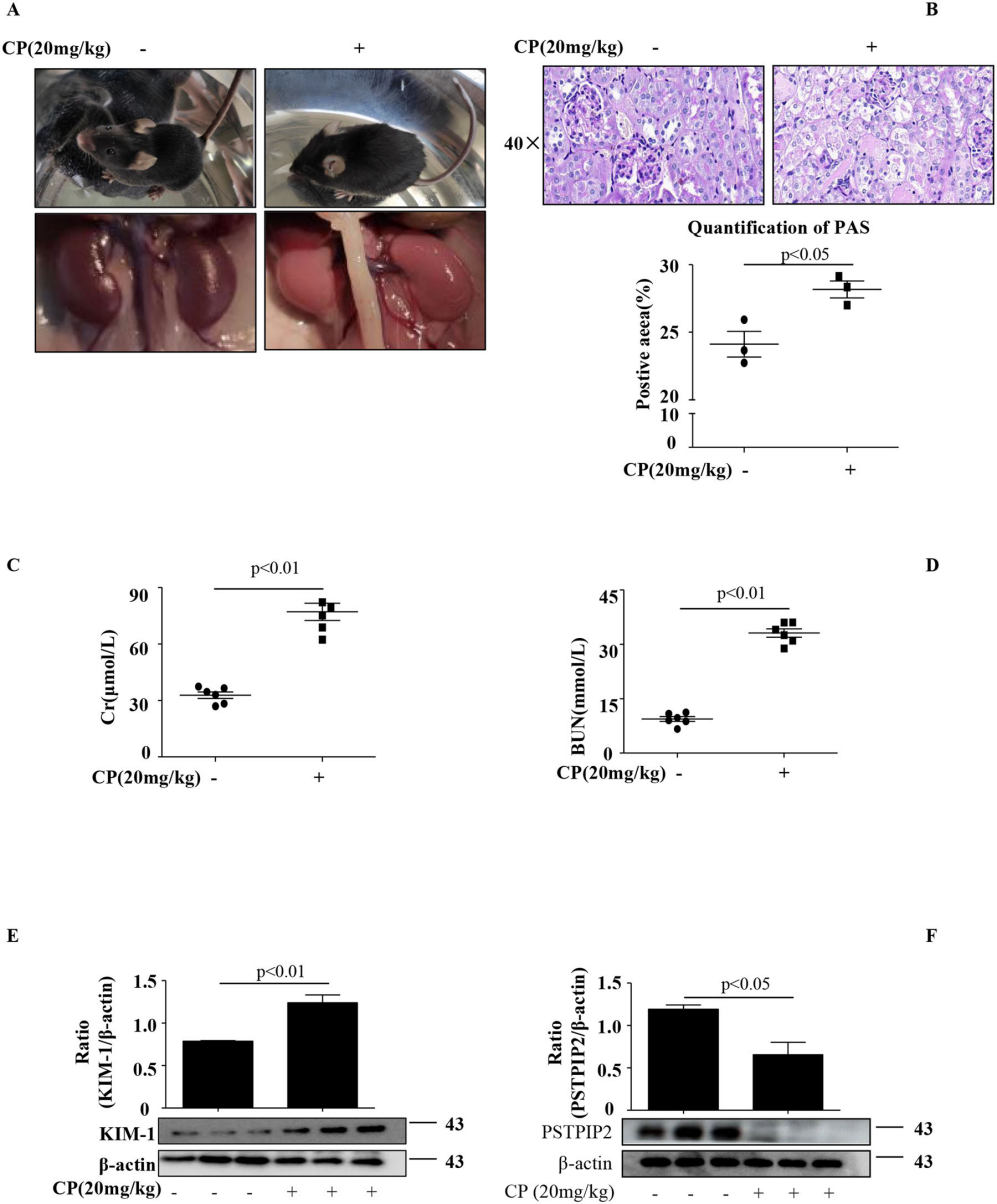
PSTPIP2 was downregulated in CP-induced AKI. **a** Representative macroscopic appearance of the kidney. **b** Kidney tissues stained with periodic acid-Schiff and quantification of renal tubular damage. **c** Serum creatinine (Cr) assay. **d** Blood urea nitrogen (BUN) assay. **e** Expression of KIM-1 in kidney tissue was detected by western blot. **f** PSTPIP2 protein levels in kidney tissue were analysed by western blot. Similar results were obtained in 3 independent experiments with 10 mice per group.

**Fig. 2:**
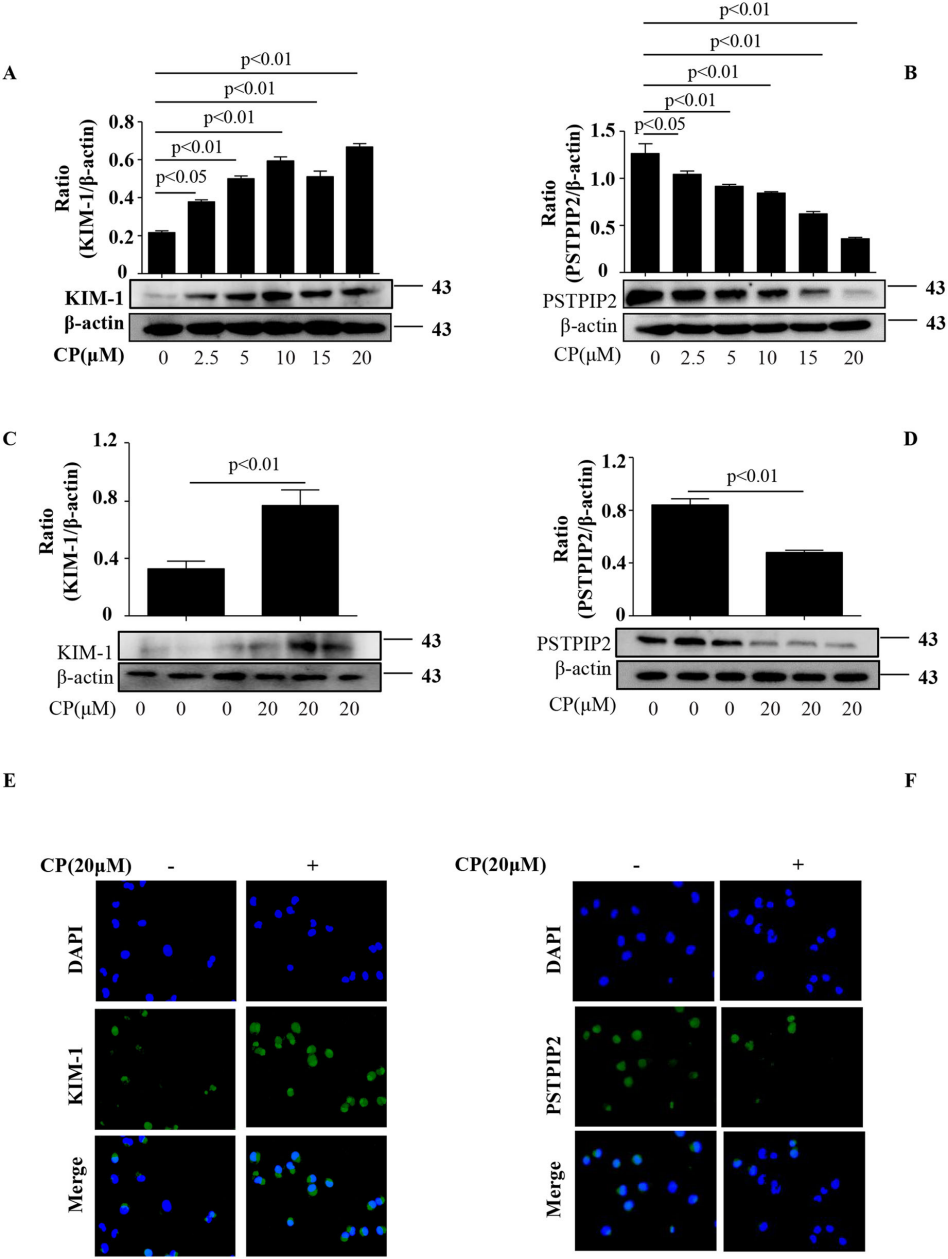
PSTPIP2 was downregulated in CP-treated HK-2 cells. **a** Effects of various concentrations of CP on the expression of KIM-1 in HK-2 cells. **b** Effects of various concentrations CP on the expression of PSTPIP2 in HK-2 cells. **c** Effect of 20 μM CP on the expression of KIM-1 in HK-2 cells detected by western blot. **d** Effect of 20 μM CP on the expression of PSTPIP2 in HK-2 cells detected by western blot. **e** Effect of 20 μM CP on the expression of KIM-1 in HK-2 cells analysed by immunofluorescence staining. **f** Effect of 20 μM CP on the expression of PSTPIP2 in HK-2 cells analysed by immunofluorescence staining. Similar results were obtained in triplicate culture assays.

To explore the role of PSTPIP2 in cisplatin-induced acute kidney injury (AKI) model, AKI mice model was initially validated by assessing the effects of cisplatin on the renal injury markers and the levels of PSTPIP2 on the third day after intraperitoneal injection^21^. Histopathological changes are direct indicators of kidney injury. The mice had messy fur and slow movements on the third day after the CP injection. After CP treatment, the macroscopic kidney appeared whitened in mice (Fig. 1a). Periodic acid-Schiff (PAS) staining showed significantly higher tissue damage in CP-treated mice compared with that in the vehicle group; the damage was characterised by loss of brush border, loss of renal tubule cells, and mould formation (Fig. 1b). Moreover, the serum creatinine (Cr) and blood urea nitrogen (BUN) levels were significantly increased in the CP group (Fig. 1c, d). These results suggest that CP induced significant lesions. Western blot analysis showed that KIM-1 (kidney injury molecule-1) was upregulated in the kidneys of the CP group (Fig. 1e). The protein level of PSTPIP2 in the kidney tissue of the CP group was decreased (Fig. 1f). Renal tubular epithelial cells were reported as the main site of cell injury during cisplatin nephrotoxicity. Then, we used HK-2 cells to induce acute kidney injury in vitro. CP at 2.5, 5, 10, 15 and 20 μM concentrations was used to determine the optimal concentration of cisplatin. As shown in Fig. 2a, b, the protein levels of KIM-1 and PSTPIP2 were increased and decreased, respectively, in a dose-dependent manner after the treatment with cisplatin. Therefore, the concentrations of cisplatin in the subsequent experiment in HK-2 cells were 20 μM. Western blot analysis showed that KIM-1 is significantly upregulated in CP-treated HK-2 cells (Fig. 2c). Moreover, western blot results demonstrated that the expression of PSTPIP2 was downregulated in CP-treated HK-2 cells (Fig. 2d). Similar patterns of KIM-1 and PSTPIP2 expression were demonstrated by immunofluorescence staining (Fig. 2e, f). Collectively, these results suggest that PSTPIP2 expression is decreased in cisplatin-treated mice and in cisplatin-treated HK-2 cells.

### PSTPIP2 administration suppressed cisplatin-induced kidney dysfunction

**Fig. 3:**
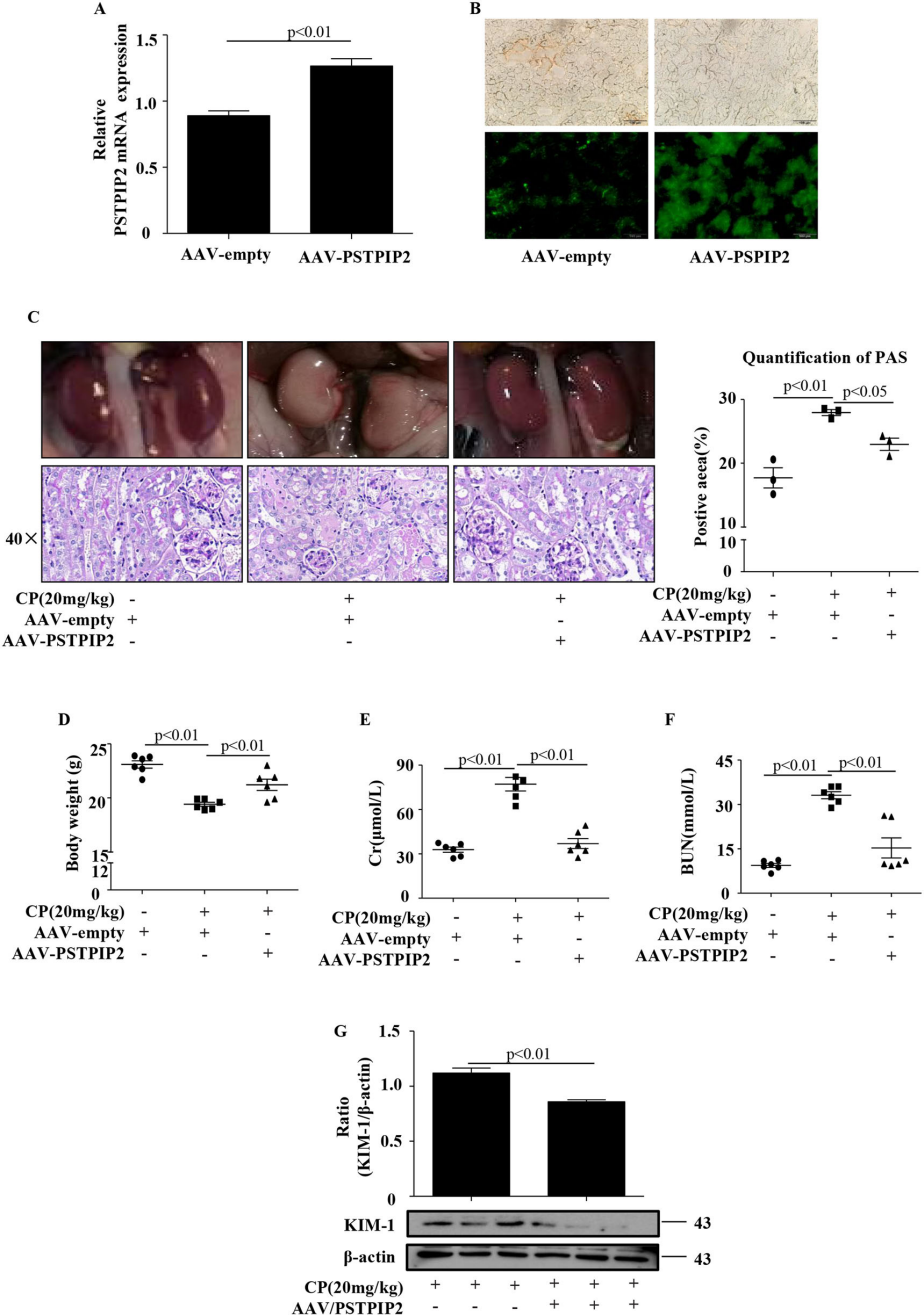
PSTPIP2 administration suppressed CP-induced kidney dysfunction. **a** PSTPIP2 mRNA levels in kidney tissue analysed by quantitative real-time PCR. **b** Representative efficient transfection of rAAV9–PSTPIP2–eGFP in kidney tissue detected by fluorescence microscopy. **c** Representative macroscopic appearance of the kidney. Kidney tissues were stained with periodic acid-Schiff and renal tubular damage was quantified. **d** Body weight in mice 72 h after CP injection. **e** Serum creatinine (Cr) assay. **f** Blood urea nitrogen (BUN) assay. **g** The protein levels of KIM-1 were detected by western blot. Similar results were obtained in 3 independent experiments with 10 mice per group.

To examine the functional importance of PSTPIP2 in the progressive kidney injury in a mouse model of AKI, rAAV9–PSTPIP2 was confirmed as an efficient means of delivery in CP-treated AKI mice (Fig. 3a, b). Macroscopically, rAAV9–PSTPIP2 can significantly improve renal function, which is related to improved preservation of kidney morphology. Histological analysis with periodic acid-Schiff (PAS) staining revealed a significant decrease in the tissue damage in the rAAV9–PSTPIP2 group compared to that in the CP group (Fig. 3c). Body weight in the rAAV9–PSTPIP2 group was slightly increased compared to that in the CP group and was close to that in the normal group (Fig. 3d). Consistently, an increase in the Cr and BUN levels was significantly attenuated by rAAV9–PSTPIP2 (Fig. 3e, f). Western blot analysis showed that KIM-1 protein expression was significantly upregulated in the CP treatment group, and rAAV9–PSTPIP2 can reduce the expression of KIM-1 protein (Fig. 3g). These results indicate that PSTPIP2 has a protective effect on CP-induced AKI.

### PSTPIP2 suppressed cisplatin-induced kidney apoptosis in vivo

**Fig. 4:**
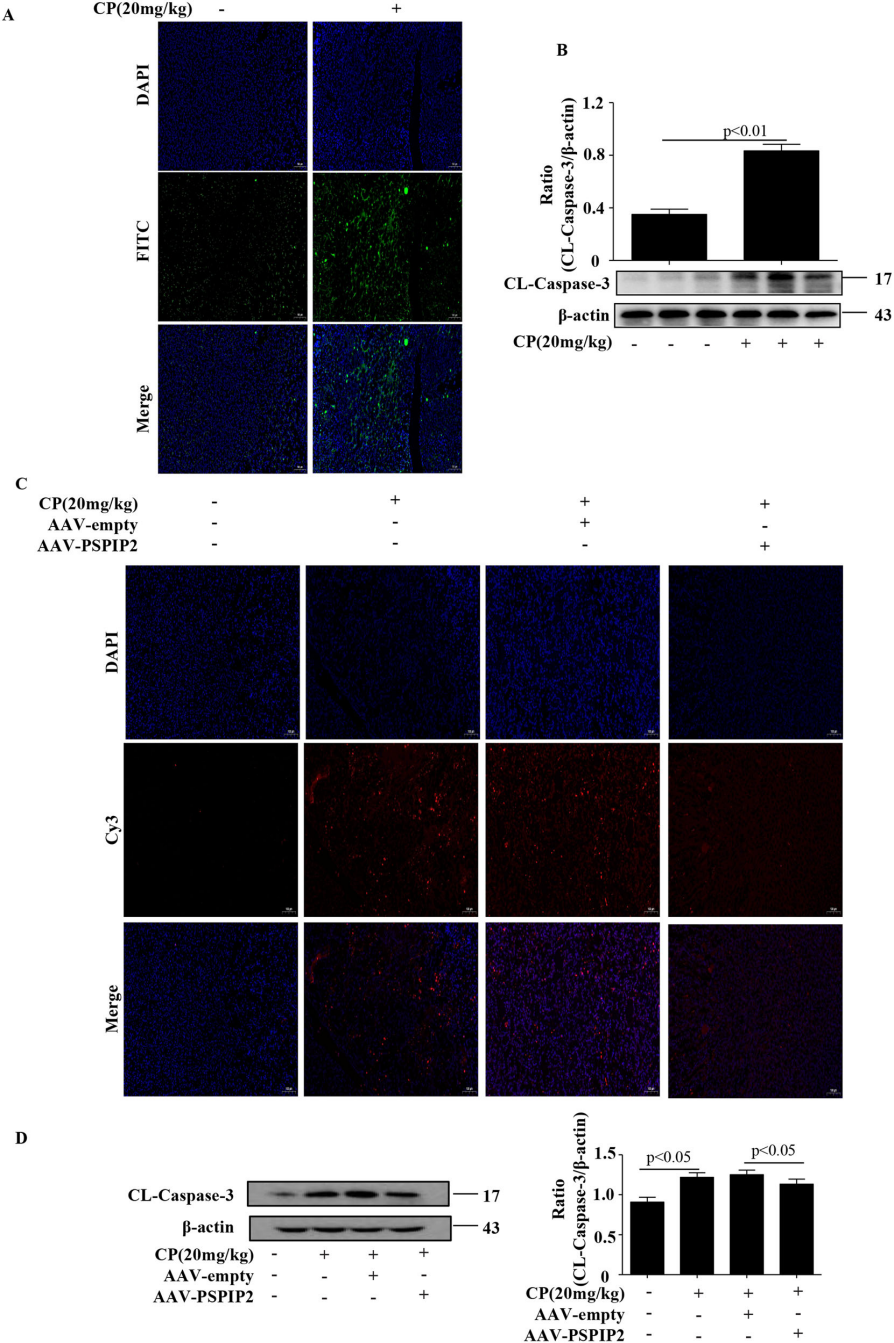
PSTPIP2 suppressed CP-induced kidney apoptosis in vivo. **a** Representative images of TUNEL staining in various groups. Scale bar, 100 μM; magnification, 10×. **b** The cleaved caspase-3 protein levels in the kidney tissue analysed by western blot. **c** Representative images of TUNEL staining in various groups. Scale bar, 100 μM; magnification, 10×. **d** The cleaved caspase-3 protein levels in various groups analysed by western blot. Similar results were obtained in 3 independent experiments with 10 mice per group.

Considerable evidence indicates that apoptosis is a significant characteristic of AKI caused by nephrotoxic medications. As shown in Fig. 4a, higher number of the TUNEL-positive cells was observed in the CP-treated group compared with that in the vehicle group. Expression of cleaved caspase-3 protein, which plays a central role in the execution phase of cell apoptosis, was clearly upregulated in the CP group compared with that in the vehicle group (Fig. 4b). To investigate the anti-apoptotic effect of PSTPIP2 in CP-induced AKI, in vivo animal experiments were performed using adeno-associated virus (AAV). Administration of rAAV9–PSTPIP2 significantly decreased the number of the TUNEL-positive cells compared with that in the CP treatment group (Fig. 4c). Furthermore, the level of cleaved caspase-3 protein was significantly downregulated in the rAAV9–PSTPIP2 administered group compared with that in the CP-treated group (Fig. 4d).

### PSTPIP2 suppressed apoptosis of renal tubular epithelial cells in vitro

**Fig. 5:**
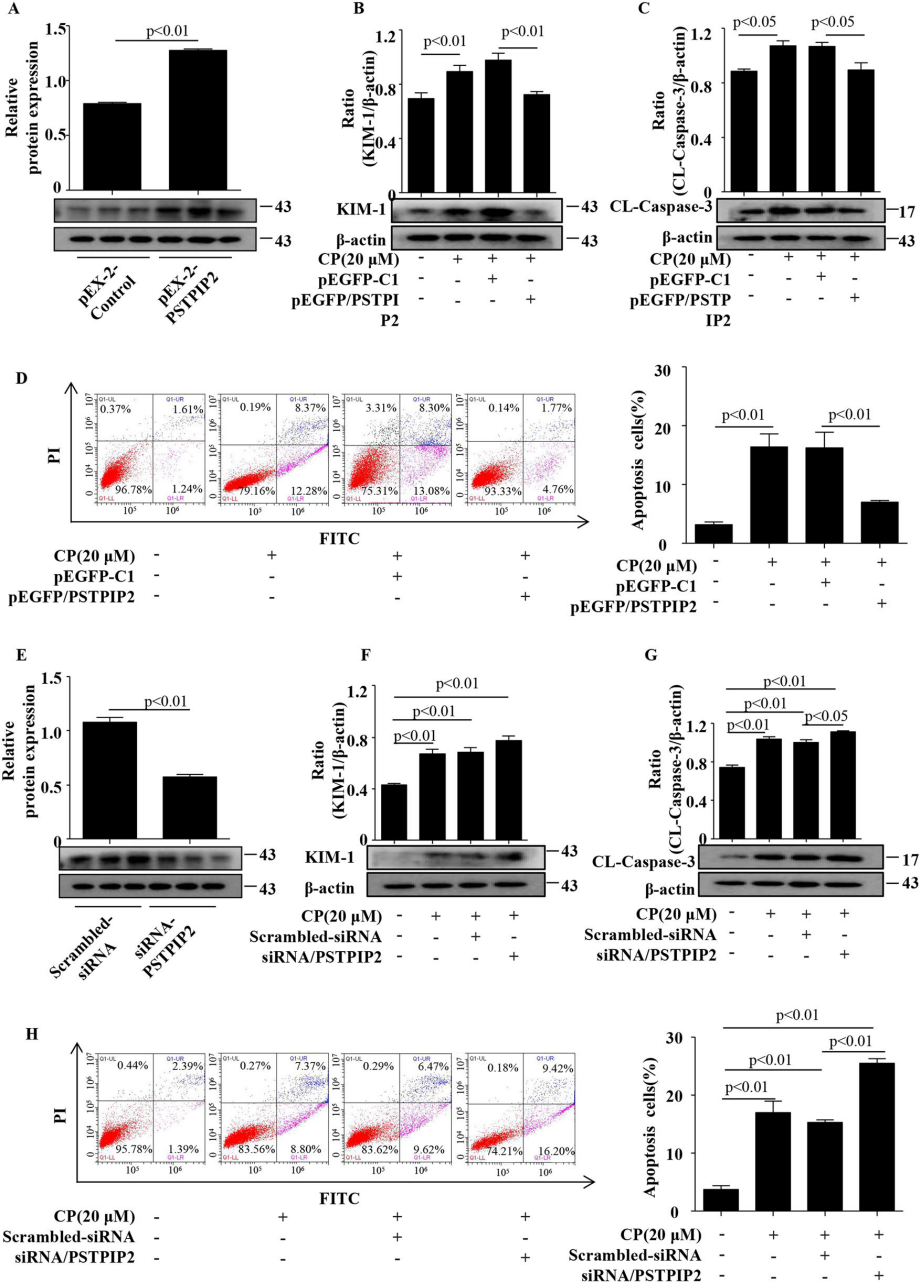
PSTPIP2 inhibited apoptosis of renal tubular epithelial cells in vitro. **a** The efficiency of PSTPIP2 overexpression in HK-2 cells detected by western blot. **b** Protein level of KIM-1 in HK-2 cells treated with pEX-2-PSTPIP2 analysed by western blot. **c** Protein level of cleaved caspase-3 in HK-2 cells treated with pEX-2-PSTPIP2 analysed by western blot. **d** Flow cytometry assay. **e** The efficiency of PSTPIP2 knockdown in HK-2 cells detected by western blot. **f** Protein level of KIM-1 in HK-2 cells treated with siRNA-PSTPIP2 analysed by western blot. **g** Protein level of PSTPIP2 in HK-2 cells treated with siRNA-PSTPIP2 analysed by western blot. **h** Flow cytometry assay in various groups. Similar results were obtained in triplicate culture assays.

To better characterise the specific role of PSTPIP2 in renal tubular epithelial cells, overexpression of PSTPIP2 was induced by transfection with the PSTPIP2 plasmid (pEX-2-PSTPIP2) in the CP-treated HK-2 cells. As shown in Fig. 5a, pEX-2-PSTPIP2 plasmid elevated PSTPIP2 protein expression. Overexpression of PSTPIP2 significantly decreased KIM-1 expression compared with that in the cells transfected with the control plasmid (Fig. 5b). The level of cleaved caspase-3 was significantly downregulated in CP-treated HK-2 cells transfected with pEX-2-PSTPIP2 (Fig. 5c). Flow cytometry analysis showed that overexpression of PSTPIP2 significantly decreased the number of apoptotic cells compared with that in the cells treated with the control plasmid (Fig. 5d). Furthermore, high efficiency siRNA-PSTPIP2 that inhibit PSTPIP2 expression were transfected into HK-2 cells to ascertain the role of PSTPIP2 in the functional alterations observed in CP-treated HK-2 cells. Western blot results indicated that siRNA-PSTPIP2 can successfully silence the expression of PSTPIP2 (Fig. 5e). Interestingly, western blot analysis showed that knockdown of the PSTPIP2 expression increased the level of KIM-1 (Fig. 5f). The level of cleaved caspase-3 was significantly upregulated in CP-treated HK-2 cells transfected with siRNA-PSTPIP2 (Fig. 5g). Flow cytometry analysis (FCM) was performed to clarify the involvement of PSTPIP2 in the apoptotic effects. In this case, siRNA-PSTPIP2 was used to confirm the effect of PSTPIP2 on the progress of apoptosis in HK-2 cells (Fig. 5h). Hence, PSTPIP2 mainly functions by inhibiting apoptosis of HK-2 cells in AKI.

### HDAC inhibitor upregulated PSTPIP2 expression in vivo and in vitro

**Fig. 6:**
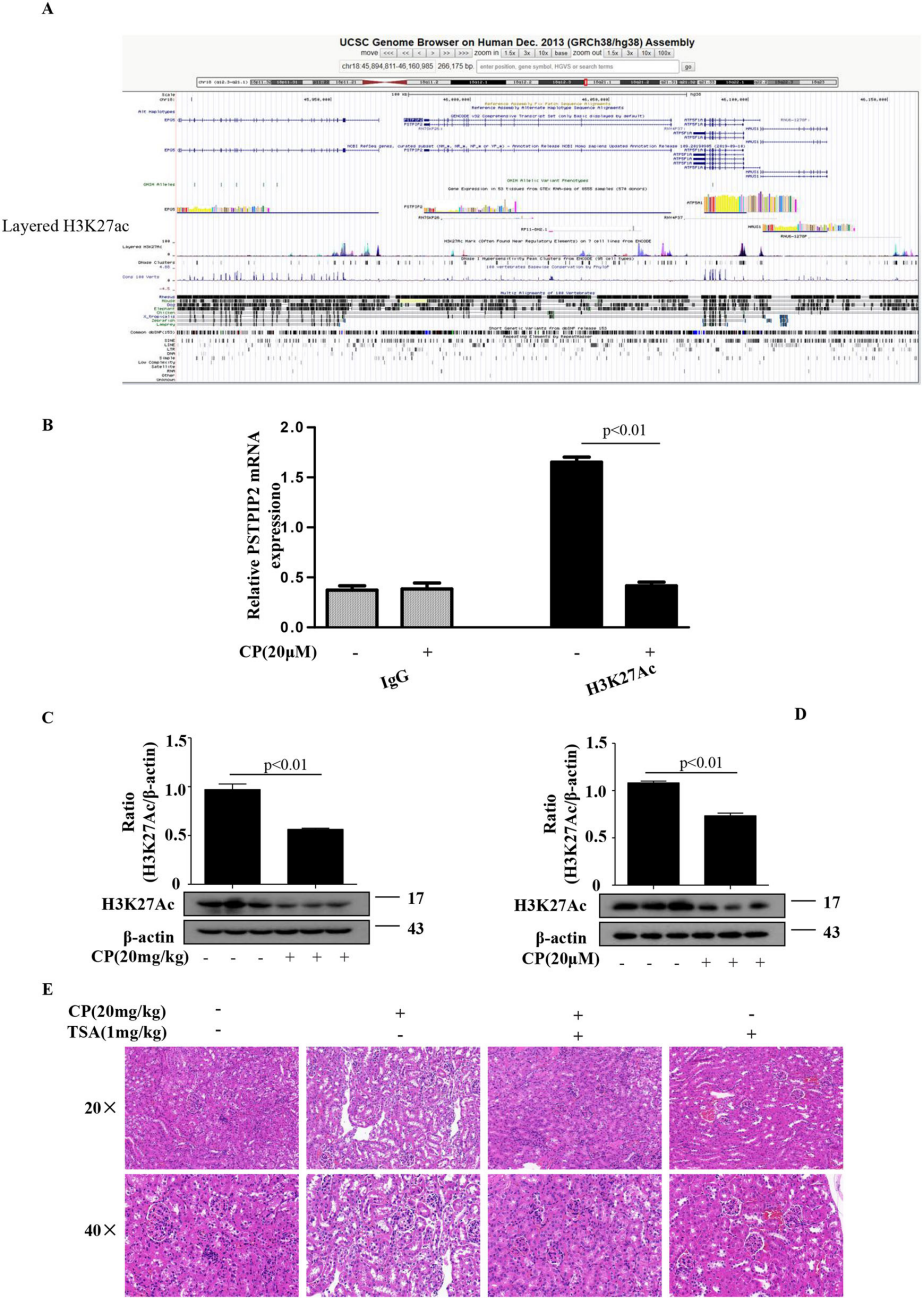
HDAC inhibitor upregulated PSTPIP2 expression in vivo and in vitro. **a** The annotation information of H3K27Ac locus from the UCSC genome browser. **b** Chromatin immunoprecipitation assay of acetylated histone in the PSTPIP2 promoter region. **c** Protein expression of H3K27Ac in kidney tissue detected by western blot. **d** Protein expression of H3K27Ac in CP-treated HK-2 cells detected by western blot. **e** Representative images of H&E staining of the kidney sections with CP-induced acute kidney injury (AKI) in mice treated with TSA. Scale bars, 50 μM and 20 μM; magnification, 20× and 40×. Similar results were obtained in 3 independent experiments with 10 mice per group or in triplicate culture assays.

**Fig. 7:**
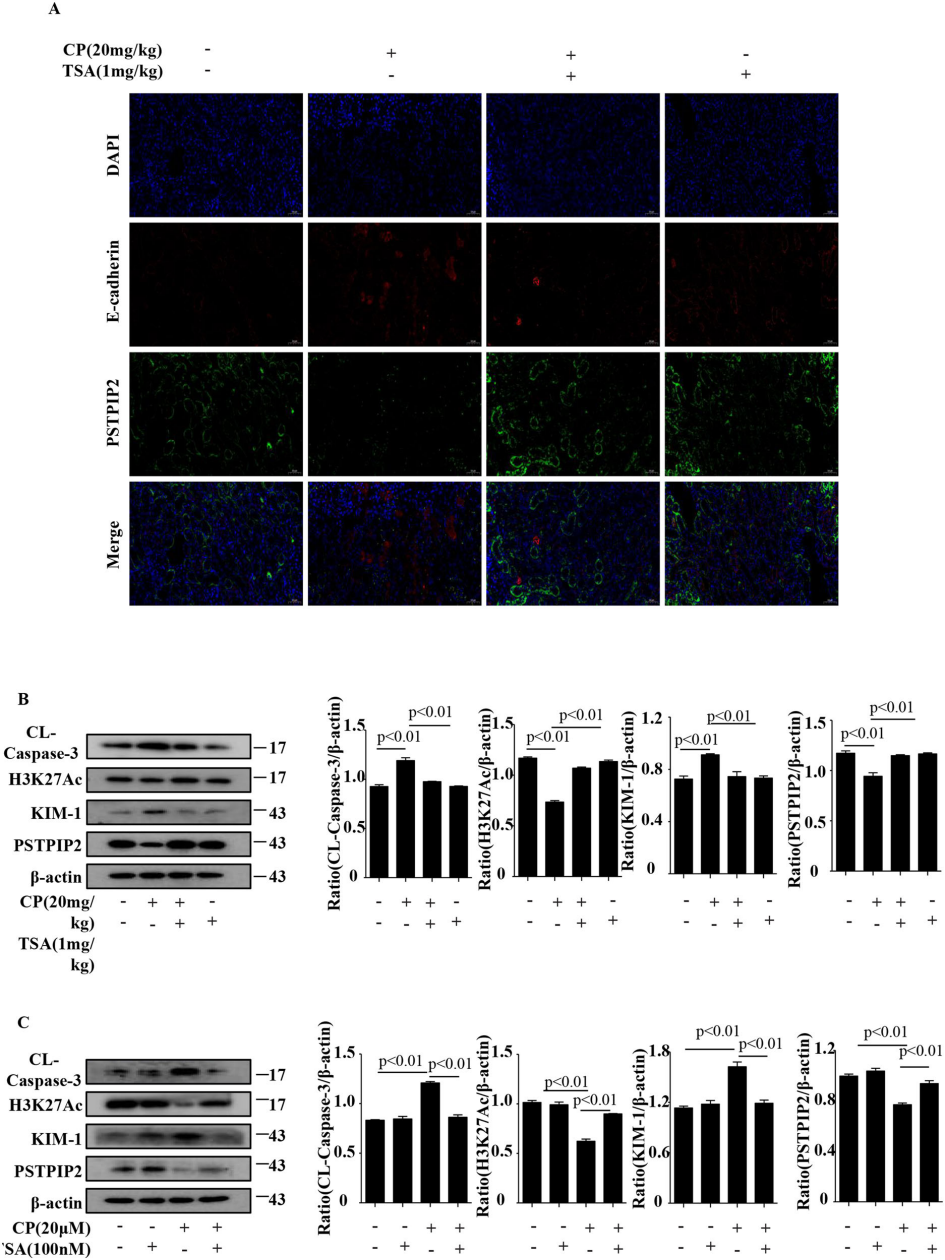
HDAC inhibitors protect from AKI by regulating the expression of PSTPIP2. **a** Immunohistochemical localisation of PSTPIP2 expression in the kidney tissues. Scale bar, 50 μM; magnification, 20×. **b** The expression of cleaved caspase-3, H3K27Ac, KIM-1 and PSTPIP2 detected by western blot in CP-treated AKI mice administered with TSA. **c** The expression of cleaved caspase-3, H3K27Ac, KIM-1 and PSTPIP2 detected by western blot in CP-treated HK-2 cells treated with TSA. Similar results were obtained in 3 independent experiments with 10 mice per group or in triplicate culture assays.

We investigated the epigenetic mechanism of PSTPIP2 regulation. The data of the UCSC genome browser include the enrichment sites for epigenetic marks (Fig. 6a). Chromatin immunoprecipitation (ChIP) assay detected that cisplatin treatment reduced histone acetylation in the promoter region of the PSTPIP2 gene (Fig. 6b). To understand the role of histone modifications in regulation of PSTPIP2, the occupancy of the histone acetylation marks (H3K27Ac) was analysed by western blot. The protein level of H3K27Ac was downregulated in CP-treated kidney tissue and in CP-treated HK-2 cells (Fig. 6c, d). Recent studies suggested that HDAC inhibitors can protect against cisplatin nephropathy^22,23^. As shown in Fig. 6e, haematoxylin and eosin (H&E) staining showed that renal tubular necrosis, mould formation and brush border retention were decreased after TSA treatment compared with that in the CP group. Localisation studies demonstrated that CP treatment decreased the expression of PSTPIP2 in tubular epithelial cells, and PSTPIP2 expression was increased in the presence of TSA (Fig. 7a). Additionally, the level of H3K27Ac protein was downregulated in vivo and in vitro, and TSA increased the protein level. The expression of KIM-1 and cleaved caspase-3 protein was downregulated in the groups treated with CP and TSA compared with that in the CP group (Fig. 7b, c).

## Discussion

This study provides a new perspective on the role of PSTPIP2 in AKI and identified a potential mechanism of PSTPIP2 inhibition of epithelial apoptosis to alleviate the nephrotoxicity of cisplatin. Administration of AAV-PSTPIP2 effectively suppressed cisplatin nephrotoxicity in mice. Interestingly, the acetylation of histones in the promoter region of PSTPIP2 gene was decreased in cisplatin-treated HK-2 cells, and HDAC inhibitor protected against cisplatin-induced AKI by promoting the expression of PSTPIP2.

Cisplatin is an effective chemotherapeutic agent used to treat a wide variety of solid tumours. However, cisplatin damages the kidney, and there is no available treatment to prevent nephrotoxicity of cisplatin^24–26^. A combination of cisplatin with HDAC inhibitors has been tested in several chemotherapy-resistant cancers to enhance the efficacy of cisplatin^27–30^. HDAC inhibitors do not have any reported toxicity towards the kidney in humans^31^. Recent studies suggested that HDAC inhibitors can protect against AKI. However, the mechanism of this effect remains unknown^23,32^.

PSTPIP2 plays a crucial role in auto-inflammatory disease, PSTPIP2 protein is expressed at a high level in macrophages and macrophage-derived cells and plays an important role in macrophage morphology and movement^13,18,33^. Auto-inflammatory disease caused by PSTPIP2 mutations is characterised by sterile inflammatory lesions in the bone and by various degrees of skin and paw inflammation in mice. Moreover, Yang et al. demonstrated that PSTPIP2 can ameliorate the degree of liver fibrosis and hepatic inflammation in CCl_4_-induced hepatic fibrosis. In this study, the expression of PSTPIP2 was decreased in AKI induced by cisplatin, and overexpression of PSTPIP2 in vivo can inhibit renal injury induced by cisplatin.

Apoptosis of renal tubular epithelial cells is a known prominent pathological feature of cisplatin-induced AKI. Inhibition of apoptosis of renal tubular epithelial cells was confirmed to reduce cisplatin nephrotoxicity. Cisplatin-induced AKI is characterised by increased apoptosis of renal tubular epithelial cells, and the results of the present study demonstrated that PSTPIP2 suppresses epithelial cell apoptosis. Consistent with these data, we demonstrate that the number of TUNEL-positive cells was significantly increased after CP injection, and AAV-PSTPIP2 can reduce the number of TUNEL-positive cells. In vitro experimented show that overexpression of PSTPIP2 suppresses apoptosis of cisplatin-treated tubular epithelial cell in agreement with the vivo data. Furthermore, the expression of cleaved caspase-3 was increased by CP treatment, and overexpression of PSTPIP2 significantly suppressed the level of cleaved caspase-3. These findings suggest that PSTPIP2 may reduce nephrotoxicity of cisplatin by inhibiting apoptosis.

Significant recent progress in the understanding of the involvement of histone modifications in gene regulation demonstrated that these modifications play a role in normal cell physiology and pathology^34,35^. The levels of histone acetylation play a crucial role in chromatin remodelling and regulation of gene transcription^36^. The presence of acetylated lysine in the histone tails is associated with a more relaxed chromatin state and activation of gene transcription, whereas deacetylation of the lysine residues is associated with a more condensed chromatin state and transcriptional gene silencing^37^. The UCSC database indicated the presence of the acetylation sites in the promoter region of PSTPIP2. Chromatin immunoprecipitation (ChIP) assay demonstrated that cisplatin decreases acetylation of histones in the promoter region of the PSTPIP2 gene. Thus, inhibition of deacetylase activity can promote PSTPIP2 gene transcription. Western blot results showed that the expression of H3K27Ac protein was decreased in CP-treated mice and HK-2 cells. HDAC inhibitors were demonstrated to suppress cisplatin-induced tubular epithelial cell apoptosis. This experiment confirms that an HDAC inhibitor can promote the expression of PSTPIP2 and inhibit apoptosis of epithelial cells.

In summary, this study is the first to show that HDAC activity suppresses PSTPIP2 gene expression in epithelial cells and HDAC inhibition enhances PSTPIP2 expression. PSTPIP2 administration suppressed kidney injury in response to cisplatin treatment, suggesting that PSTPIP2 can be a part of a useful therapeutic strategy for treatment of cisplatin nephrotoxicity in humans. Further studies are needed to determine the mechanism of PSTPIP2-mediated suppression of epithelial cell apoptosis.

## Materials and methods

### Materials and reagents

Trichostatin A (TSA) and cisplatin (CP) were purchased from Sigma Aldrich (St. Louis, MO, USA). CP was used in the form of 1 mg/ml solution in sterile normal saline. The primary antibodies against β-actin, KIM-1, and PSTPIP2 and goat anti-rabbit IgG HRP secondary antibodies were purchased from Bioss (Beijing, China). Primary antibodies against cleaved caspase-3 and H3K27Ac were obtained from Abcam (Cambridge, UK). A creatinine (Cr) assay kit and a blood urea nitrogen (BUN) assay kit were purchased from Jiancheng Bioengineering Institution (Nanjing, China). An Annexin V-FITC/PI apoptosis detection kit (BB-4101) was obtained from BestBio (Shanghai, China).

### Model of cisplatin-induced AKI

Six- to eight-week-old male C57BL/6 J mice weighing 18–22 g were obtained from the Experimental Animal Center of Anhui Medical University. All animal procedures were reviewed and approved by the Institutional Animal Experimental Ethics Committee. All mice were housed in a comfortable environment and were adaptively maintained for a week before the experiment. Mice were administered cisplatin (1 mg/ml solution in sterile normal saline) at 20 mg/kg or normal saline in a single intraperitoneal (i.p.) injection. Mice were sacrificed 72 h after cisplatin injection. The blood samples and kidney tissues were collected for further analysis.

### Adeno-associated virus 9 mouse model

The adeno-associated viruses were developed and obtained from Hanheng (Shanghai, China). All mice were randomly divided into 6 groups (*n* = 12 per group): vehicle group, CP group, AAV9 group, AAV9 + CP group, AAV9-PSTPIP2 group, and AAV9- PSTPIP2 + CP group. Vehicle group mice were administered normal saline in a single i.p. injection. CP group mice were administered CP at 20 mg/kg in a single i.p. injection. Adeno-associated virus 9 (AAV9) group mice were injected with an empty AAV9 vector by tail vein injections and normal saline was administered in a single i.p. injection. AAV9 + CP group mice were injected with an empty AAV9 vector by tail vein injections and CP was administered at 20 mg/kg in a single i.p. injection. AAV9-PSTPIP2 group mice were injected with an AAV9- PSTPIP2 vector by tail vein injections and normal saline was administered in a single i.p. injection. AAV9- PSTPIP2 + CP group mice were injected with an AAV9- PSTPIP2 vector by tail vein injections and CP was administered at 20 mg/kg in a single i.p. injection. Animals were sacrificed 72 h after cisplatin injection, and samples of blood and kidney tissues were collected for further analysis.

### Cell culture

Human kidney tubular epithelial cell line (HK-2) was provided by Prof. Huiyao Lan (Li Ka Shing Institute of Health Science, Hong Kong, China). HK-2 cells were cultured in DMEM/F12 (HyClone, Logan, UT, USA) supplemented with 5% (v/v) foetal bovine serum (FBS; Merck Millipore, Darmstadt, Germany). HK-2 cells were incubated at 37 °C with 5% CO_2_.

### Creatinine (Cr) and blood urea nitrogen (BUN) assay kits

Blood samples were collected for creatinine and BUN measurements according to the manufacturer’s instructions.

### Histopathology

Kidney tissue of mice was fixed in 4% paraformaldehyde for 48 h immediately after the sacrifice, and the tissue samples were embedded in paraffin. Sections (5 μm thick) were stained with a periodic acid Schiff (PAS) staining kit. The PAS sections were observed and imaged using light microscopy.

### Immunofluorescence staining

Renal tissue sections of mice were blocked with 5% bovine serum albumin (BSA) at 37 °C for 30 min to avoid non-specific staining. Sections were incubated with anti-PSTPIP2 (1:500) and anti-E-cadherin (1:500) overnight at 4 °C. Then, the sections were incubated with a secondary antibody (1:100) in the dark at 37 °C for 1 h. The stained sections were examined by inverted fluorescence microscopy. HK2 cells were incubated overnight at 4 °C with antibodies against PSTPIP2 (1:500) and KIM-1(1:500) followed by goat anti-rabbit IgG for 1 h at room temperature. Cells were counterstained with DAPI (Beyotime Biotechnology, China) and visualised by fluorescence microscopy.

### Western blot

Proteins from the kidney tissue (30 mg) and HK-2 cells were extracted with RIPA lysis buffer (Beyotime Biotechnology, China). Proteins were separated using 10 or 12% sodium dodecyl sulphate-polyacrylamide gel electrophoresis (SDS-PAGE) and transferred to PVDF membranes (Millipore Corp, Billerica, MA, USA). The PVDF membranes were blocked with 5% skim milk for 2 h at room temperature and washed three times with TBST. Subsequently, the PVDF membranes were incubated with primary antibodies against PSTPIP2 (1:1,000), KIM-1 (1:1000), cleaved -caspase-3 (1:1000) or H3K27Ac (1:1000) overnight at 4 °C followed by incubation with secondary antibodies (1:10,000) for 1 h at room temperature. The protein bands were visualised with an ECL kit (ECL-plus, Thermo Scientific, Pittsburgh, PA, USA).

### TUNEL assay

Apoptosis in renal tissue was measured by the TUNEL assay using one step TUNEL apoptosis assay kit according to the manufacturer’s protocol (Kegen Biotech, China). TUNEL-positive cells (red) were identified by fluorescence microscopy.

### Flow cytometry

The extent of the programmed cell death was examined using an Annexin-V-FITC apoptosis detection kit (BestBio, China). HK-2 cells were trypsinized and collected from the suspension by centrifugation. The cells were resuspended in 400 μl of Annexin V binding buffer and stained with 5 µl Annexin V-FITC and 10 µl PI in the dark. Apoptosis of the cells was detected using a flow cytometer (Beckman, USA) within 1 h, and the data were analysed using FlowJo 7.6 software (TreeStar, USA).

### RNA interference analysis

Small interfering RNA (siRNA) oligonucleotides against the PSTPIP2 gene or scrambled sequences were designed and synthesised by Hanheng (Shanghai, China). The siRNA sequences were as follows:

PSTPIP2-siRNA (sense, 5′-GGUCAGUGUAGUUGAUGUA-3′ and antisense, 5′-UACAUCAACUACACUGACC-3′); scrambled siRNA (sense, 5′-UUCUCCGAACGUGUCACGUTT-3′ and antisense, 5′-ACGUGACACGUUCGGAGAATT-3′). HK-2 cells were transfected with 1,000 ng/ml PSTPIP2-siRNAs or scrambled-siRNAs using the Lipofectamine 2000 transfection reagent (Invitrogen, USA) according to the manufacturer’s instructions. After 6 h, Opti-MEM was replaced by DMEM/F-12, and the cells were treated with CP for 24 h. The silencing efficiency was determined by western blot.

### Transfection with PSTPIP2 plasmid

HK-2 cells were seeded in 6-well plates. After attachment to the well, HK-2 cells were transfected with 1,000 ng/ml pEX-2-PSTPIP2 or pEX-2-Control plasmids by using Lipofectamine 2000 transfection reagent (Invitrogen, USA) and Opti-MEM for 6 h according to the manufacturer’s instructions. Then, Opti-MEM was changed to DMEM/F-12 containing 5% FBS, and the cells were treated with CP for 24 h. The transfection efficiency was determined by western blot.

### Chromatin immunoprecipitation (ChIP) assay

HK-2 cells were stimulated with CP (20 μM) and CP (20 μM) combined with TSA. The corresponding control cells were cultured to 80–90% confluence. ChIP assays were performed using a SimpleChIP® enzymatic chromatin IP kit with magnetic beads (#9003, Cell Signaling Technology, USA) according to the manufacturer’s instructions. The following antibodies were used to immunoprecipitate the crosslinked protein-DNA complexes: rabbit anti-H3K27Ac and normal rabbit IgG. The immunoprecipitated DNA was purified for PCR analyses with primers specific for the putative binding sites within the promoter of PSTPIP2.

### Statistical analysis

Statistical significance was determined after computing single factor ANOVA and/or unpaired two-tailed Student’s *t*-test. Data error bars correspond to ± standard error of the mean (SEM). All experiments included ≥3 biological repeats as indicated in the figure legends; the number of the technical replicates is stated for each method. Statistical analyses were performed using the GraphPad Prism 5.0 software (USA). *P* < 0.05 was considered statistically significant.
